# Monthly Summary

**Published:** 1894-04

**Authors:** 


### Monthly Summary.

REPAIRINF VULCANITE PLATES.—Dr. J. S. Latimer, in the *Cosmos*, draws attention to the sometimes forgotten fact that it is not necessary to make undercuts in repairing vulcanite plates; that if the surfaces are freshened and painted over with a solution of the unvulcanized caoutchouc, the new material will unite with it. Some one told him that it would answer as well to wet the freshened (scraped) surfaces with kerosene.

Dr. Day showed a novel method of expediting the packing of cases. First apply shellac varnish to the cast, allow it to harden, then add the vulcanite in pieces of from a half inch square or larger, pressing each down on the cast, and smoothing down prominences with a warm spatula until the whole surface is covered, and the building up around the teeth completed as usual. Then flask at one pouring and vulcanize.

Dr. Latimer. after shellacking, covers the surface of the cast with No. 20 tin foil, burnished smooth. Dip a piece of rubber in chloroform and paint over all the surface of the tin.—*Dominion Dental Journal.*

---

LUBRICATE YOUR DISKS AND STRIPS.—By C. N. Johnson.—We see so many dentists still using sandpaper disks and strips in finishing fillings without vaseline or oil to lubricate them, that we feel like once more calling attention to this matter. It is a censurable species of inhumanity to run a disk dry on a gold filling, or to see-saw a strip back and forth between teeth without using a lubricant. A disk or strip will not heat up so rapidly if covered with vaseline, and contrary to prevalent opinion, the cutting properties will be improved. Quite often the question is asked by those who never use a lubricant: "But will not the vaseline or oil prevent the sandpaper from cutting?" A careful test of the matter will prove to these men that their impressions are wrong. If there were no other recommendations for the use of lubricants than the one of increased cutting power, this would be a sufficient inducement to always employ them. When disks are used with the rubber dam in place, it is extremely difficult to prevent a dry disk from catching up the dam in its revolutions and tearing it, but a disk well lubricated will play over the dam with little danger of a mishap. Another recommendation lies in the fact that when well smeared with vaseline, a disk becomes flexible and can be pressed into depressions and be made to cut at any desired point by guiding it with an instrument. This is well-nigh impossible when a dry disk is used.

This latter consideration is very important wherever a disk is employed between teeth for finishing a filling. If a proper contour is to be left to the filling, it is necessary that the disk shall cut only at the cervical portion of the filling and not at the contact point. A flexible disk may be pressed against the neck of the tooth if desired and all the cutting confined to that region, but a dry disk will almost invariably cut away the contact point and make a flat filling. The variety of curves to be given a flexible disk in cutting is limited only by the dexterity of the operator. Another argument in favor of lubricating the disk or strip is one of economy. In this condition it will hold the fine particles of gold on its surface, and the dentist who preserves his old disks and strips for a time and then turns them over to a refiner for melting, will be surprised at the result.—*Dental Review.*

---

AN INTERESTING CASE.—A young lady, about seventeen years of age, called for treatment. She was suffering from severe pain in the region of the right superior lateral incisor. In her own words, "suffering from neuralgia in the front teeth on the right side," and wished the lateral incisor extracted, as it was loose and had caused her trouble enough. We found the teeth in remarkably good condition for a girl of this age, excepting the tooth mentioned, it being slightly loosened; we also found a slight bluish line running through the gum over the root of this tooth and part way across the adjoining central incisor. We learned from her mother that about five years previous to her visit she fell, striking the gum here with the point of a lead pencil, but both the girl and her mother, in response to our questions, felt sure that the pencil was all drawn out at the time. But we found the lower border of the process absorbed over the root of the lateral incisor, and a small piece of the pencil lying directly over the root; also another piece, possibly a quarter of an inch in length, that had been forced endwise between the central and lateral incisors. It was so tightly imbedded that we did not remove it in one piece, but found it necessary to dissect it out, a small piece at a time. The wound was then dressed and the patient dismissed. She was not heard from for several weeks, when the report was made that the "loose tooth had grown tight and the neuralgia had disappeared."—*International.*

PARALYSIS CURED BY LANCING THE GUMS, ON A LITTLE GIRL OF TWENTY MONTHS.—The little one showed fever and diarrhœa, which the mother thought came from the teeth. A physician being called in, prescribed for the fever and for the diarrhœa. After two days the child became paralyzed in the left leg, and suffered with an inactivity of the kidneys, for which nitre was prescribed with beneficial effect. This, however, lasted only two days. Dr. Smith found none of the teeth had erupted, and reported the fact to the physician, who said he could not see any possible connection between that fact and the disease of the child. Another physician being called in, thought the condition might be caused by the teeth. Dr. Smith requested the privilege of lancing the gums. He made a deep incision over the cuspids on the right side. The second day the child could stand on its foot, and the third day could walk, and now there is nothing apparently wrong, except a slight numbness in the foot.—*Dental Cosmos.*

---

FITTING BANDS TO ROOTS.—Slight imperfections of this band are probably present oftener than most crown makers imagine, and every precaution should be taken to avoid them. A good practice to follow when the crown is finally adjusted is to go around the lower margin of the band with a burnisher, or, what is better, where it can be used, a plugger point, and gently tap the edge of the band to the snuggest possible fit. It will sometimes be found that the extreme edge of the band will yield slightly under the force of the plugger and hug the root of the tooth a hair-breadth closer, even when it has been supposed that the best possible fit had previously been obtained. The gain of even the thickness of a piece of note paper in the close adaptation of a band to a root may mean much in the final outcome of the operation.—*Items of Interest.*

